# Construction of yeast one-hybrid library and screening of transcription factors regulating LhMYBSPLATTER expression in Asiatic hybrid lilies (*Lilium* spp.)

**DOI:** 10.1186/s12870-021-03347-1

**Published:** 2021-11-29

**Authors:** Yuwei Cao, Mengmeng Bi, Panpan Yang, Meng Song, Guoren He, Jing Wang, Yue Yang, Leifeng Xu, Jun Ming

**Affiliations:** Institute of Vegetables and Flowers, Chinese Academy of Agricultural Sciences, Beijing, 100081 China

**Keywords:** *Lilium*, Anthocyanins, Transcriptional factor, Yeast one-hybrid library, *LhMYBSPLATTER*

## Abstract

**Background:**

Anthocyanins, which belong to flavonoids, are widely colored among red-purple pigments in the Asiatic hybrid lilies (*Lilium* spp.). Transcription factor (TF) LhMYBSPLATTER (formerly known as LhMYB12-Lat), identified as the major kernel protein, regulating the anthocyanin biosynthesis pathway in ‘Tiny Padhye’ of Tango Series cultivars, which the pigmentation density is high in the lower half of tepals and this patterning is of exceptional ornamental value. However, the research on mechanism of regulating the spatial and temporal expression differences of *LhMYBSPLATTER*, which belongs to the R2R3-MYB subfamily, is still not well established. To explore the molecular mechanism of directly related regulatory proteins of LhMYBSPLATTER in the anthocyanin pigmentation, the yeast one-hybrid (Y1H) cDNA library was constructed and characterized.

**Results:**

In this study, we describe a yeast one-hybrid library to screen transcription factors that regulate *LhMYBSPLATTER* gene expression in *Lilium*, with the library recombinant efficiency of over 98%. The lengths of inserted fragments ranged from 400 to 2000 bp, and the library capacity reached 1.6 × 10^6^ CFU of cDNA insert, which is suitable to fulfill subsequent screening. Finally, seven prey proteins, including BTF3, MYB4, IAA6-like, ERF4, ARR1, ERF WIN1-like, and ERF061 were screened by the recombinant bait plasmid and verified by interaction with the *LhMYBSPLATTER* promoter. Among them, ERFs, AUX/IAA, and BTF3 may participate in the negative regulation of the anthocyanin biosynthesis pathway in *Lilium*.

**Conclusion:**

A yeast one-hybrid library of lily was successfully constructed in the tepals for the first time. Seven candidate TFs of *LhMYBSPLATTER* were screened, which may provide a theoretical basis for the study of floral pigmentation.

**Supplementary Information:**

The online version contains supplementary material available at 10.1186/s12870-021-03347-1.

## Background

Lily (*Lilium* spp.), a worldwide ornamental flower, has a significant range of flower colors, mainly composed of anthocyanins. Anthocyanins are among the secondary metabolites that contribute to the colors of tepals. Anthocyanins play important roles in UV-B protection, pathogen, and biotic stress defenses, attracting pollinators and seed-dispersing animals [1–4]. Additionally, anthocyanins in the tepals of flowers among cultivars is the important horticultural characteristic that directly determines the aesthetic and commercial value of floricultural crops [5, 6]. Spatially and temporally distinct anthocyanin accumulation in tepals of lilies leads to various colour patterning, including bicolor and spots (raised spots and splatter-type spots), such as the Asiatic hybrid lilies Tango Series cultivars ‘Tiny Padhye’, in which anthocyanins are specifically colored in the basal tepals (referred to as splatter-type spots) and have special ornamental value [6]. *LhMYB12-Lat* which derived from the Asiatic hybrid lilies cv. ‘Tiny Padhye’ causing splatter-type spots, was renamed *LhMYBSPLATTER* (*LhMYBSPL*) because it was not an allele gene of *LhMYB12* [7]. LhMYBSPLATTER, the kernel of the MBW transcriptional complex that was composed of MYB, bHLH and WD40 transcription factors (TFs), regulated the structural genes in splatter-type anthocyanin biosynthesis [8] and transport pathways [9] in Tango Series cultivars of Asiatic hybrid lilies. Thus, LhMYBSPLATTER is a crucial transcription factor among various pathways that regulates pigmentation at splatter-type spots in lilies.

In the plants, anthocyanins accumulation during flower development is induced by environmental (exogenous) and developmental (endogenous) factors, including stress-response factors (BBX, HY5, NAC, and WRKY) [10–15], certain growth hormone-response factors (ERF, ARF/IAA, and bZIP) [16–26], and nutritional factors (BT2 and LBD) [21, 27, 28]. Among them, the target *MYB* gene expression may be controlled by response factors based on DNA *cis*-elements, which caused differential anthocyanin contents and coloring.

The yeast one-hybrid system is generally used to screen prey protein interactions based on bait DNA promoter sequence and analyze transcriptional regulation controlled by TFs that bind to DNA *cis*-elements located in the gene promoters [29]. Therefore, candidate prey proteins could be obtained by yeast one-hybrid assay, such as in wheat [30], *Arabidopsis* [31, 32], *Populus* [33], tobacco [34], *Cymbidium* [35] etc. Nevertheless, until now, the TFs acting on the *LhMYBSPLATTER* gene promoter that positively/negatively responds to anthocyanin biosynthesis and accumulation remains unclear in flower plants.

LhMYBSPLATTER, a positive transcription factor, was shown to regulate pigmentation at splatter-type spots in the Asiatic hybrid lilies Tango Series cultivars, and its expression was consistent with that of anthocyanin structural genes [8, 36]. However, the mechanism by which upstream regulation leads to differential expression of *LhMYBSPLATTER* has rarely been studied. Therefore, the present study aimed to screen the prey interaction proteins and determine the TFs specifically directly binding to the *LhMYBSPLATTER* gene promoter based on a yeast one-hybrid system. The findings of this study may provide a further understanding of the regulatory pathway of anthocyanin in lily.

## Results

### Cloning of *LhMYBSPLATTER* promoter

**Fig. 1:**
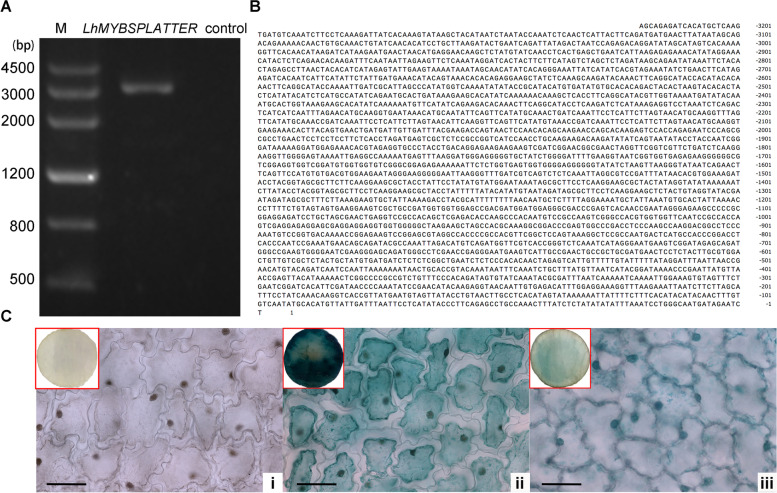
Cloning of *LhMYBSPLATTER* promoter and GUS expression levels in the disc of lily tepals. **A** Electrophoretic diagram of cloned *LhMYBSPLATTER* promoter. **B** Sequencing result of cloned *LhMYBSPLATTER* promoter. **C** GUS histochemical staining detected in the disc of tepals transformed by negative control (i), *35S* (ii) and *LhMYBSPLATTER* promoter sequences (iii); bars = 250 μm

The lily *LhMYBSPLATTER* promoter was isolated based on amplifying promoter sequences primers using the genome walking method and submitted as MW719044. The PCR products, a 3222 bp length, were verified by agarose gel electrophoresis (Fig. 1a) and shown in Fig. 1b. The *cis*-elements and transcription factor binding sites showed ABA-, auxin-, MeJA-, gibberellin-, ethylene-, light-, and stress-responsive elements by online software PlantCARE and PlantPAN 2.0 in *LhMYBSPLATTER* promoter sequence (Table 1).

**Table 1:** Promoter *cis*-elements prediction

Element	Sequence	Position	Function
A-box	CCGTCC	− 912 to − 907	cis-acting regulatory element
ABRE	ACGTG	− 2726 to − 2722	ABA-responsive element
ATCT-motif	AATCTAATCC	− 3154 to − 3144	part of a conserved DNA module involved in light-responsiveness
ARR	CAAATCT	− 447 to − 441	ARR motif
Box II	ACACGTAGA	− 1882 to − 1874	part of a light-responsive element
Box 4	ATTAAT	− 216 to − 211	light-responsive element
CAAT-box	CAAT	−98 to − 95	common cis-acting element in promoter and enhancer regions
CAT-box	GCCACT	− 935 to − 930	cis-acting regulatory element related to meristem expression
CGTCA-motif	CGTCA	− 1587 to − 1583	cis-acting regulatory element involved in the MeJA-responsiveness
G-box	CACGTG	− 1513 to − 1508	cis-acting regulatory element involved in light-responsiveness
GATA-motif	GATAGGA	− 198 to − 192	part of a light-responsive element
GC-motif	CCCCCG	− 1107 to − 1102	enhancer-like element involved in anoxic specific inducibility
GCC-box	CCGCCGTC	− 376 to − 369	ethylene-responsive element
GCN4_motif	TGAGTCA	− 821 to − 815	cis-regulatory element involved in endosperm expression
GT1-motif	GGTTAA	− 507 to − 502	light-responsive element
HSE	AGAANNTTCT	− 2869 to − 2861	HSF element
LAMP-element	CTTTATCA	− 2467 to − 2460	part of a light-responsive element
LTR	CCGAAA	− 792 to − 787	cis-acting element involved in low-temperature responsiveness
MBS	CAACTG	− 3091 to − 3086	MYB binding site involved in drought-inducibility
Myb	CAACTG	− 470 to − 465	Myb motif
MYC	CATGTG	− 2540 to − 2539	MYC element
P-box	CCTTTTG	− 3005 to − 2999	gibberellin-responsive element
Sp1	GGGCGG	− 1673 to − 1668	light-responsive element
TATA-box	TATA	−30 to −27	core promoter element around − 30 of transcription start
TCA-element	CCATCTTTTT	− 1896 to − 1887	cis-acting element involved in salicylic acid responsiveness
TCCC-motif	TCTCCCT	− 1116 to − 1110	part of a light-responsive element
TGA-element	AACGAC	− 1818 to − 1813	auxin-responsive element
TGACG-motif	TGACG	−1587 to −1583	cis-acting regulatory element involved in the MeJA-responsiveness
W box	TTGACC	− 2561 to − 2556	WRKY motif

### GUS activity assay

To verify whether the promoter of *LhMYBSPLATTER* could impact the expression of the *LhMYBSPLATTER* gene, we constructed *proLhMYBSPLATTER*::GUS recombination plasmid to infect the disc of lily tepals. The promoter activity can be directly reflected by GUS staining. As shown in Fig. 1c, GUS staining results showed that the *LhMYBSPLATTER* promoter could activate gene expression of *LhMYBSPLATTER* in lily tepals.

### Construction of a cDNA library

The Asiatic hybrid lily ‘Tiny Padhye’ upper tepals and bases of tepals from the S2 stage were collected for total RNA extraction. The quality of the total RNA samples is shown in Fig. S1, showing the bands corresponding to the intact 28S and 18S rRNA. The total RNAs had an A_260_/A_280_ ratio of 2.06–2.10 and a concentration of 480–556 ng/μL, which fulfilled the conditions for constructing a cDNA library.

**Fig. 2:**
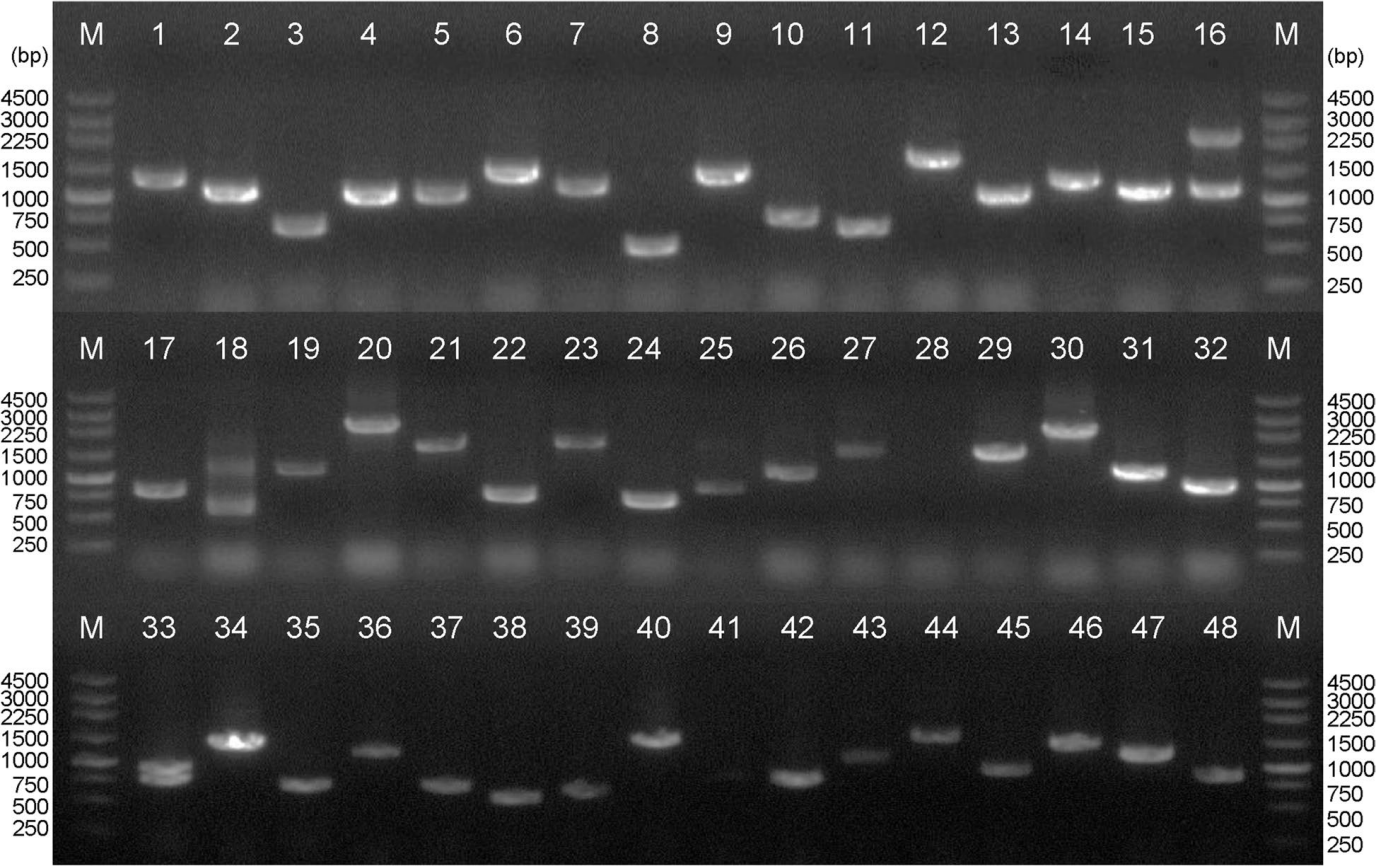
Agarose gel electrophoresis of positive, healthy colonies sequencing to quantify the cDNA library from randomly selected 48 colonies

Agarose gel electrophoresis showed that the cDNA library was successfully constructed, and the primary library volume was above 1.6 × 10^6^ CFU, with a recombination efficiency of more than 98%. The homogenization results verified that the redundancy rate was 1% by sequencing 96 single positive clones (Fig. 2).

### Construction of bait-reporter strains and determining AbA concentration

**Fig. 3:**
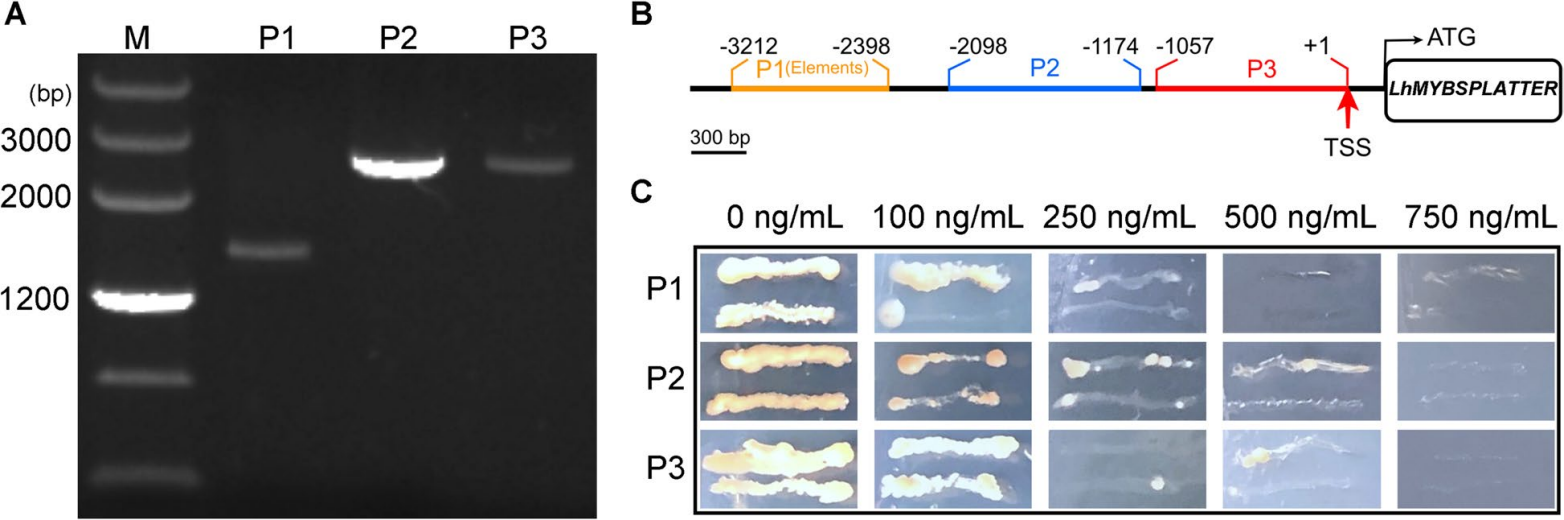
Construction of bait-yeast strains and minimum concentrations of AbA for inhibiting self-activation. **A** Agarose gel for reconstructing bait-reporter strains; M: Marker; P1: *LhMYBSPLATTER*-P1(elements); P2: *LhMYBSPLATTER*-P2; P3: *LhMYBSPLATTER*-P3. **B** Schematic diagram of *LhMYBSPLATTER* promoter fragments. **C** The minimum inhibitory concentration of AbA

As shown in Fig. 3a, the colony PCR results were consistent with the expected size of PCR products (1.35 kb plus the inserted fragment), identifying correct integration into yeast cells.

To exclude yeast transcription factors for target sequence recognition, we measured minimum concentrations of AbA for inhibiting self-activation. As shown in Fig. 3b-c, basal expression of pAbAi-*LhMYBSPLATTER*-P1(elements), pAbAi-*LhMYBSPLATTER*-P2 and pAbAi-*LhMYBSPLATTER*-P3 bait strains were 250 ng/mL, 500 ng/mL, and 500 ng/mL, respectively.

### Screening of a yeast one-hybrid library

pAbAi-*LhMYBSPLATTER*-P1(elements), pAbAi-*LhMYBSPLATTER*-P2, and pAbAi-*LhMYBSPLATTER*-P3 yeast cell suspensions obtained on SD/−Leu plates were diluted to 1/100, and 124, 77, and 20 clones, respectively. The number of screened clones were 1.9 × 10^6^, 1.2 × 10^6^, and 0.3 × 10^6^, respectively.

### Extraction of the prey plasmids and confirming positive interactions

The positive colonies of pAbAi-*LhMYBSPLATTER*-P1(elements), pAbAi-*LhMYBSPLATTER*-P2 and pAbAi-*LhMYBSPLATTER*-P3 were selected from the SD/−Leu plates, 149, 140, and 141 clones, respectively, and grow on SD/−Leu brown media to extract the prey plasmids. Then, the extracted plasmids were transferred with prey strains to the SD/−Leu medium containing the appropriate concentration of AbA, and the pGADT7 plasmid was used as a negative control. The positive plasmids of the growing yeast strains were identified that regulate the expression of the *LhMYBSPLATTER* gene by interacting with *cis-*elements in the *LhMYBSPLATTER* promoter. Finally, the confirmed sequences of lily were compared by Blastx (https://blast.ncbi.nlm.nih.gov/Blast.cgi), including Zinc finger, ERF, GRP, and MYB predicted proteins, which originate from *Elaeis guineensis*, *Phoenix dactylifera*, and *Musa acuminata* (Table 2).

**Table 2:** Screening results of *LhMYBSPLATTER* promotor transcriptional factors

Position	Accession number	Biological annotation	Species
P1	AEU17861.1	heat shock transcription factor	*Lilium longiflorum*
P1	XP_012476945.1	basic transcription factor 3	*Gossypium raimondii*
P1	ASV46333.1	MYB4	*Lilium regale*
P1	XP_010913061.1	zinc finger CCCH domain-containing protein ZFN-like isoform X2	*Elaeis guineensis*
P2	XP_008810485.1	auxin-responsive protein IAA6-like isoform X1	*Phoenix dactylifera*
P2	XP_009412068.1	ethylene-responsive transcription factor 4	*Musa acuminata*
P2	XP_009387936.1	ethylene-responsive transcription factor ERF071-like	*Musa acuminata*
P2	XP_030477126.1	gibberellin-regulated protein 6	*Syzygium oleosum*
P3	XP_010905671.1	two-component response regulator ARR1	*Elaeis guineensis*
P3	XP_008804592.1	ethylene-responsive transcription factor WIN1-like	*Phoenix dactylifera*
P3	XP_010913767.1	ethylene-responsive transcription factor ERF061	*Elaeis guineensis*

### Expression analysis of candidate TFs of *LhMYBSPLATTER*

**Fig. 4:**
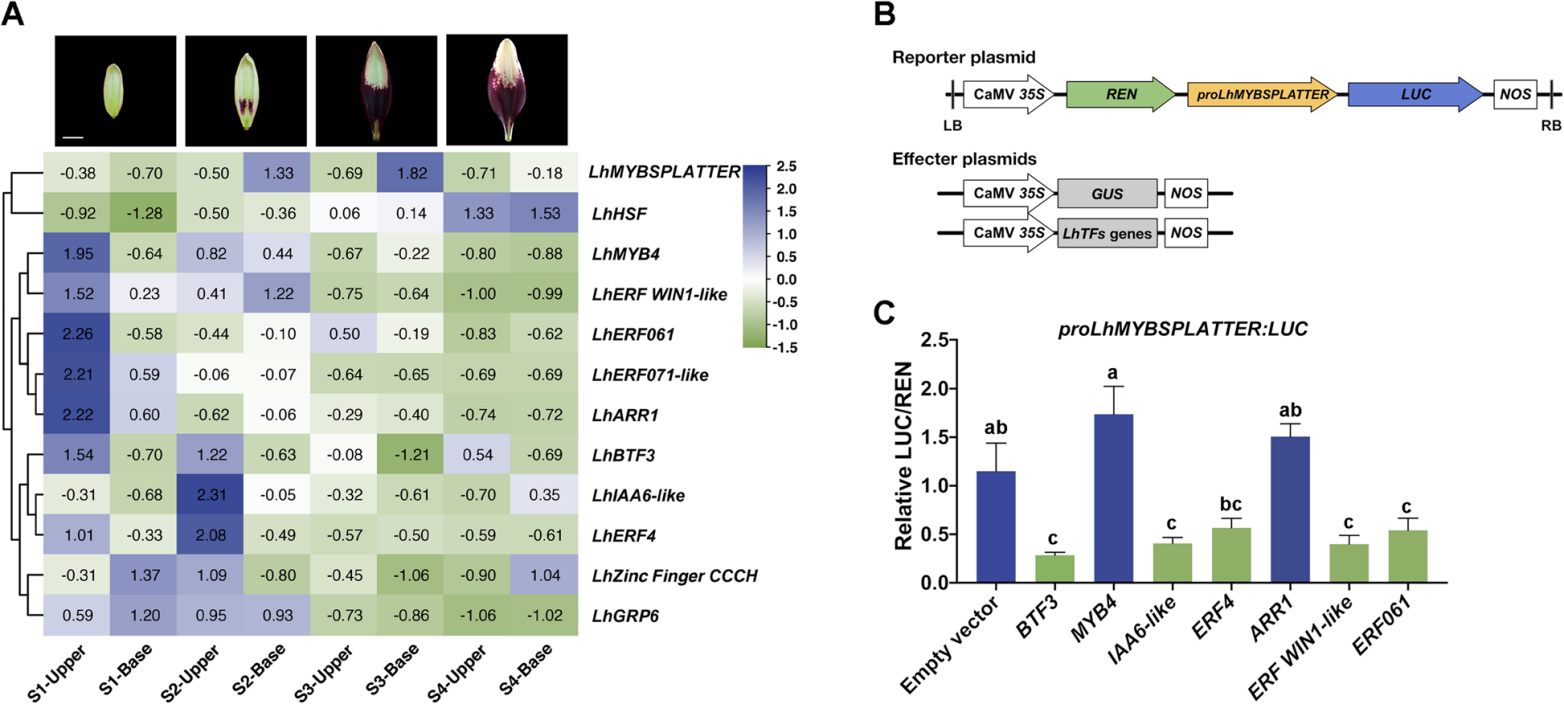
Genes expression analysis and detecting LUC/REN activity to verify candidate transcription factors co-transformation to activate the *LhMYBSPLATTER* promoter. **A** Variation of candidate genes expression in Y1H screen library. The value indicated the temporal and spatial expression of candidate genes treated with log2. **B** Schematic diagram of vectors used for the dual-luciferase assay. **C** Dual-luciferase assay showing relative *LhMYBSPLATTER* activation to the transcription factor genes obtained by screening library. Each experiment was triplicated, and the results were analyzed as average value ± SD

To verify the interaction between the expression levels of candidate TFs and *LhMYBSPLATTER*, we analyzed the expression of candidate TFs genes in ‘Tiny Padhye’ tepals by qRT-PCR. We found that expression levels of most genes tested were significantly different in the upper and basal tepals (Fig. 4a). These results suggest that the genes (*MYB4*, *ERF WIN1-like*, *ERF061*, *ERF071-like*, *ARR1*, *BTF3*, *IAA6-like*, and *ERF4*) may be involved in the suppression of *LhMYBSPLATTER*-mediated anthocyanin biosynthesis regulation in lily. However, we found the expression of *HSF*, *ZF CCCH* and *GRP6* genes were no significant differences in the upper and basal tepals, which indicated that the difference in anthocyanin contents caused by *LhMYBSPLATTER* expression level was not significantly co-related to *HSF*, *ZF CCCH* and *GRP6* genes.

### LUC reporter assay

We performed LUC reporter assays to determine how several potential TF genes regulate the activity levels of *proLhMYBSPLATTER*. As shown in Fig. 4b-c, *BTF3*, *IAA6-like*, *ERF4*, *ERF WIN1-like*, *ERF061* negative regulated *proLhMYBSPLATTER*, but *MYB4* and *ARR1* barely affected the activity level of *proLhMYBSPLATTER*. Thus, *BTF3*, *IAA6-like*, *ERF4*, *ERF WIN1-like*, *ERF061* was found to mainly suppress *proLhMYBSPLATTER* to negative regulate anthocyanin accumulation in lily.

## Discussion

The yeast one-hybrid system is commonly recognized as a useful technique for detecting new DNA-protein interactions with regulation pathways and is widely used in functional genomics studies [37, 38]. In general, the vital standards for constructing a high-quality yeast-one hybrid library include the purity, integrity and concentration of mRNA. The recombination efficiency, transformation efficiency, and library capacity, including the integral expression information, must contain at least 1 × 10^6^ CFU [35, 39]. In this study, the three indexes of the yeast library of *Lilium* were 98%, 400–2000 bp and 1.6 × 10^6^ CFU/mL of cDNA insert, respectively, which fulfilled the requirements for further library screening.

By screening a high-quality library of interactions between target prey proteins and bait plasmid links in regulatory networks enables fast and effective identification in higher organisms [31, 35, 40, 41]. In this study, we successfully constructed a high-quality cDNA library of *LhMYBSPLATTER* gene promoter that can be used for yeast one-hybrid assays, providing strong evidences that unknown proteins with functional identification of regulating anthocyanin biosynthesis in Asiatic hybrid lily. Therefore, the present study screened several potential TF genes, including various stresses and growth hormones, such as ARR1 (MW719046), HSF (MW719048), ERF WIN1-like (MW719031), IAA6-like (MW719034), and ERF4 (MW719035).

Among them, BTF3, IAA6-like, ERF4, ERF WIN1-like, ERF061, and ERF071-like may bind to the *LhMYBSPLATTER* promoter and have an inhibitory regulation to *LhMYBSPLATTER*. IAA6-like were identified from *LhMYBSPLATTER-*P2, which encoded an AUX/IAA protein containing an N terminal DNA binding domain [42]. Auxin signaling, which is crucial for normal plant growth and development, including those related to flavonoid and anthocyanin metabolism, mainly depends on ARF-Aux/IAA interactions [21, 25]. In apple, the study proved that adding NAA alone suppressed anthocyanin synthesis even at low concentrations, and the higher concentration of NAA, the more severe inhibition of anthocyanin biosynthesis [21, 25, 43]. Moreover, treating strawberry fruits with exogenous auxin can delay fruit ripening by down-regulating the expression of genes related to anthocyanin synthesis [44], which was similar to the modulation we hypothesized here. In this study, a target protein homologous to an anthocyanin-related protein in lily was screened out based on the *LhMYBSPLATTER* promoter as the bait vector, with negatively correlated expression with *LhMYBSPLATTER*. Therefore, we speculated that ARF might be involved in the transcriptional regulation of the *LhMYBSPLATTER* promoter in anthocyanin biosynthesis. Additionally, ERF TFs, a large family of TFs that feature the conserved AP2/ERF domain, play key roles in plant growth, development and various stress, including modulating anthocyanin biosynthesis [24, 45]. Recent studies have reported that MdERF was found to interact with *proMdMYB9*, *proMdMYB1* and *proMdMYB11* to promote anthocyanin and proanthocyanidin biosynthesis in apple [22, 24]. However, ethylene inhibited anthocyanin biosynthesis in red Chinese pear fruits [23]. Notably, we hypothesized that ERFs screened from the cDNA library may be involved in suppressing anthocyanin biosynthesis, which might contrast with previous studies. Therefore, the regulation mechanism of the anthocyanin biosynthesis by hormone-mediated response factors varies significantly among different species.

In addition, there are several light-responsive elements in the *LhMYBSPLATTER* promoter sequence. However, light-related TFs, such as ELONGATED HYPOCOTYL5 (HY5) [46] and WRKY family [13] proteins, have not been identified by the yeast-one hybrid library. Moreover, we found that shading the unstained bud did not affect the difference of anthocyanin in the upper and basal tepals. Therefore, we speculated that light might not be the key external factor in the differential expression of anthocyanins between the upper and bases of tepals in lily.

Expression analysis showed that MYB4 and ARR1 were significantly highly expressed in the upper tepal at S1 stage, so we hypothesized that they might have an inhibitory effect on the regulation of anthocyanin biosynthesis. However, the dual-luciferase assay found that they might be involved in the positive regulation of the anthocyanin biosynthesis pathway. Therefore, we speculated that there may be feedback regulation in lily, further verified by functional studies.

Among the candidate TFs for *LhMYBSPLATTER* promoter screened by the yeast-one hybridization, positive regulators of which expression dramatically upregulate in the basal tepals at S1 and S2 stages are somewhat regrettably not included. We speculated that it might be due to the expression of *LhMYBSPLATTER* gene began to differ significantly in the upper and basal tepals during S2 stage when a cDNA library was constructed. However, the tepals had already accumulated anthocyanin during S2 stage, leading to the failure to screen positive TFs that regulate *LhMYBSPLATTER* gene, but a large number of TFs were screened to inhibit anthocyanin accumulation in the upper tepals.

In addition, upstream of the start codon, *LhMYBSPLATTER* sequence from − 357 to − 11 and from − 163 to 1 showed high similarity to *LhMYB12-Lollypop* (DDBJ database, LC612729) and *LhMYB19S-Lollypop* (DDBJ database, LC612730) sequences, respectively. The similar region was longer between *LhMYBSPLATTER* and *LhMYB12-Lollypop* than between *LhMYBSPLATTER* and *LhMYB19S-Lollypop*. Interestingly, the coding region and 5′ terminal sequence of *LhMYBSPLATTER* derived from ‘Tiny Padhye’ is completely consistent with that of *LhMYBSPLATTER-Latvia* (NCBI database, AB827442.1) that is not allele gene with *LhMYB12-Lollypop* [7]. Thus, an ancestral sequence of *LhMYBSPLATTER*, *LhMYB12-Lollypop* and *LhMYB19S-Lollypop* were likely mutated in their promoter region, giving rise to functional difference for anthocyanin pigmentation [47].

## Conclusions

In conclusion, the identified TFs from *LhMYBSPLATTER* promoter and screened high-quality cDNA library may help get a deep insight into the regulatory pathway of anthocyanin in lily. Our results are the first to identify them in tepals, and may provide theoretical support and possible research directions for identifying the regulatory associations between potential TFs and *LhMYBSPLATTER* gene promoter. Further studies are still needed to verify their functions as anthocyanin regulators.

## Materials and methods

### Plant materials

The Asiatic hybrid lily Tango Series cultivar ‘Tiny Padhye’ were obtained commercially from Zhejiang Licai garden company limited (Zhejiang, China) and were grown in a greenhouse at the Institute of Vegetables and Flowers, Chinese Academy of Agricultural Sciences (Beijing, China). Flowers were divided into four stages (S1-S4) [8]. The flowers of inner tepals were collected at S2 stage, in which 20 tepals as one biological sample and three biological replicates for each tissue, and upper tepals and bases of inner tepals were collected separately and then frozen in liquid nitrogen and stored at − 80 °C until further use. The Oriental hybrid lily cv. ‘Sorbonne’ were used for GUS staining.

### Cloning of *LhMYBSPLATTER* promoter

Genomic DNA was isolated from ‘Tiny Padhye’ upper tepals and bases of tepals (S1-S4) using Hi-DNAsecure Plant Kit (polysaccharide & polyphenolic-rich) (TIANGEN, Beijing, China), according to the manufacturer’s instructions. The promoter of *LhMYBSPLATTER* was amplified using Genome Walking Kit (Clontech, Dalian, China) by SP1-SP3 primers (Table S1). The motif of the promoter was analyzed by online software of PlantCARE (http://bioinformatics.psb.ugent.be/webtools/plantcare/html/) and PlantPAN 2.0 (http://plantpan2.itps.ncku.edu.tw).

### Construction of the *β*-glucuronidase (GUS) expression vector

The promoter region of *LhMYBSPLATTER* was ligated to the pBI121 vector with a GUS tag, generating constructs *proLhMYBSPLATTER*::GUS plasmid using homologous recombination. The empty pBI121-GUS vector served as a positive control. The constructed vector and empty vector were introduced into *Agrobacterium tumefaciens* strain EHA105, and the *A. tumefaciens* strain was infiltrated into the disc of tepals of the Oriental hybrid lily cv. ‘Sorbonne’ with a higher anthocyanin accumulation [48]. The primers used to amplify the promoter of *LhMYBSPLATTER* with linker are listed in Table S1.

### Creating a yeast one-hybrid cDNA library

Total RNA was extracted from ‘Tiny Padhye’ upper tepals and bases of S2 stage tepals using an RNAprep Pure Plant Kit (polysaccharide & polyphenolic-rich) (TIANGEN, Beijing, China), quickly frozen in liquid nitrogen and then stored at − 80 °C until further use. A cDNA library of ‘Tiny Padhye’ was synthesized and mixed using SMART cDNA Library Construction Kit and Advantage II PCR Kit (Clontech, CA, USA). The cDNA was normalized using Trimmer Direct cDNA Normalization Kit, purified by TaKaRa MiniBEST DNA Fragment Purification Kit (Takara, CA, USA), and enriched using a CHROMA SPIN-1000-TE (Clontech, CA, USA). After enriched, cDNA was ligated into the pGADT7-SfiI vector using DNA ligation Kit, and then a normalized cDNA library of ‘Tiny Padhye’ was obtained by purification.

### Construction of bait plasmids

The promoter sequence of *LhMYBSPLATTER* was divided into P1(elements), P2 and P3, and cloned into pAbAi vector digested with SacI and XhoI, respectively. The primers used to amplify the coding sequences and the promoter region are listed in Table S1. The *LhMYBSPLATTER* promoter products with linker were purified on 1% agarose gel and ligated into the linearized bait pAbAi vector using homologous recombination. The ligated products were transformed into *E. coli* DH5α competent cells and positive recombinant clones were screened using LB media containing ampicillin.

### Transformation of linearized bait plasmids into yeast cells

The recombinant plasmids were digested with BstBI/BbsI, integrated into the yeast genome (Y1H Gold) using the PEG/LiAc method, and transferred onto solid agar synthetic defined (SD) medium -Ura and incubated for 2–3 days. The positive recombinant clones were identified by PCR using Matchmaker Insert Check PCR Mix 1 (Clontech, CA, USA). The empty pAbAi vector was identified as a positive control with a PCR product size of 1.4 kb. Then, the recombinant bait-reporter yeast strains were screened on SD/−Ura medium with optimal Aureobasidin A (AbA) concentration to suppress and selected onto each of the following media: SD/−Ura without AbA, SD/−Ura with 100 ng/mL AbA, SD/−Ura with 250 ng/mL AbA, SD/−Ura with 500 ng/mL AbA, SD/−Ura with 750 ng/mL AbA, and SD/−Ura with 1000 ng/mL AbA. The bait-reporter yeast strains were grown for 3 days at 28–30 °C, and the minimum AbA concentration that could completely inhibit strains was determined to use for further library screening.

### Screening of a Y1H library

A cDNA library (5 μg) was transformed with bait reporter yeasts. Then yeast cell was resuspended in 0.9% NaCl (approximately 15 mL) and spread 100 μL of 1/10, 1/100 dilutions on each SD/−Leu with optimal AbA concentration for 3–5 days. Afterwards, the number of colonies were calculated using the following formula: Transformation efficiency = [CFU/mL on SD/−Leu] × [dilution factor] × [resuspension volume (15 mL)].

### Confirming positive interactions and extracting the prey plasmids

Positive clones from plates of screening Y1H library were re-transferred into new SD/−Leu medium with same AbA concentration for 3–5 days incubation. To determine the positive interaction, single clones were re-streaked 2–3 times for incubation. Then genuine positive colonies used the Matchmaker Insert Check PCR Mix 2 (Clontech, CA, USA) to amplify prey library inserts. Analyze PCR products by electrophoresis on a 1.0% TBE agarose gel. The healthy generated single colonies were segregated in broth SD/−Leu media and prepared to extract the library plasmids from yeast, using TIANprep Yeast Plasmid DNA Kit (TIANGEN, Beijing, China), following the manufacturer instruction. The pGADT7-Recexpression vector (5 μL) was transformed into *E. coli* DH5α competent cells and screened using LB media containing ampicillin at 37 °C for 12 h. After culture, the plasmids from the LB medium were extracted by TIANpure Mini Plasmid Kit (TIANGEN, Beijing, China) for co-transformation and sequencing.

### Quantitative real-time PCR analysis

Total RNA was isolated from ‘Tiny Padhye’ upper and basal tepals in four stages using an RNAprep Pure Plant Kit (polysaccharide & polyphenolic-rich) (TIANGEN, Beijing, China), according to the manufacturer’s instructions. Briefly, the first-strand cDNA was synthesized using TransScript® II One-Step gDNA Removal and cDNA Synthesis SuperMix (TransGen Biotech, Beijing, China) and oligo_(dT)_ primers. The qRT-PCR reactions were performed using *Perfectstar*™ Green qPCR SuperMix (TransGen Biotech, Beijing, China) and a Bio-Rad CFX96 system. The relative expression level of quantification was calculated based on the 2^−∆∆Ct^ formula method [49]. *LilyActin* was used as an internal control [50]. The primers for RT-qPCR were synthesized by Sangon Biotech (Shanghai, China; Table S1).

### Dual-luciferase transient expression assay

The promoter sequence of *LhMYBSPLATTER* was recombined into CP516-LUC plasmid as reporter and TFs obtained from the screening library were inserted into pCAMBIA3301 plasmid as an effector. The effector and LUC reporter for normalization were mixed and transformed into the tobacco leaves by *Agrobacterium*-mediated transient expression for transient transfection. After co-infected with 2–3 days in the normal grown condition at room temperature, the LUC and REN values were determined using a GloMax® 20/20 Luminometer (Promega, USA). A Dual-Luciferase Reporter Gene Assay Kit (Yeasen, Shanghai, China) was used to detect the LUC/REN activity. The relative LUC activity was measured from the ratio of LUC to REN values. Each experiment was triplicated and the results were analyzed as average value ± SD.

## Supplementary Information

**Additional file 1: Table S1.** The list of primers used in this study.

**Additional file 2: Figure S1.** Total RNA extracted from flowers tepals at S2 stage. The line 1 indicated the upper tepals and the line 2 indicated basal tepals.

## Data Availability

The sequence datasets generated and/or analyzed during the current study are available in the NCBI repository, https://www.ncbi.nlm.nih.gov/nuccore/MW719044.1/, https://www.ncbi.nlm.nih.gov/nuccore/MW719048.1/, https://www.ncbi.nlm.nih.gov/nuccore/MW719030.1/, https://www.ncbi.nlm.nih.gov/nuccore/MW719038.1/, https://www.ncbi.nlm.nih.gov/nuccore/MW719043.1/, https://www.ncbi.nlm.nih.gov/nuccore/MW719034.1/, https://www.ncbi.nlm.nih.gov/nuccore/MW719035.1/, https://www.ncbi.nlm.nih.gov/nuccore/MW719037.1/, https://www.ncbi.nlm.nih.gov/nuccore/MW719039.1/, https://www.ncbi.nlm.nih.gov/nuccore/MW719046.1/, https://www.ncbi.nlm.nih.gov/nuccore/MW719031.1/, https://www.ncbi.nlm.nih.gov/nuccore/MW719032.1/. The data set supporting the results of this article are included in the article and Additional files.

## Abbreviations

TFs: Transcription factors
Y1H: Yeast one-hybrid
BTF3: Basic transcription factor 3
ERF: Ethylene response factor
GRP: Gibberellin-regulated protein
qRT-PCR: Real-time quantitative PCR
HY5: ELONGATED HYPOCOTYL 5
