# Supplementary figures and images for: Construction of yeast one-hybrid library and screening of transcription factors regulating LhMYBSPLATTER expression in Asiatic hybrid lilies (Lilium spp.)
Source: BMC Plant Biol. 2021 Nov 29;21:563. doi: 10.1186/s12870-021-03347-1 (PMC8628396; doi:10.1186/s12870-021-03347-1)

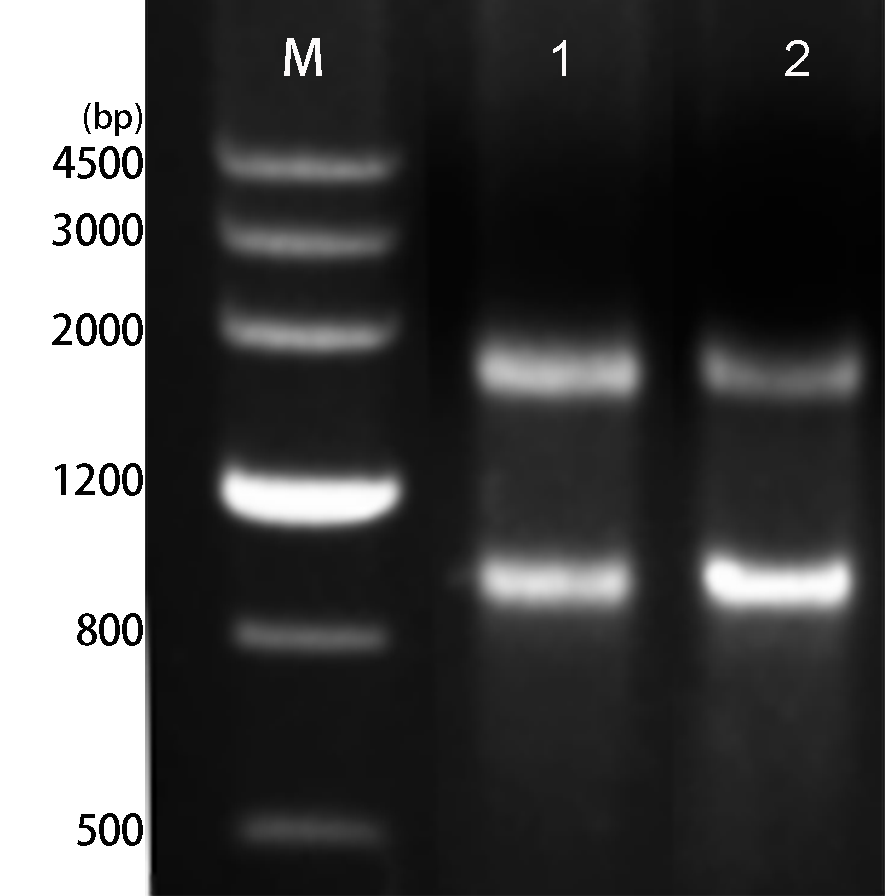

Supplement: Supplementary file 2 — Additional file 2: Figure S1. Total RNA extracted from flowers tepals at S2 stage. The line 1 indicated the upper tepals and the line 2 indicated basal tepals. [file 12870_2021_3347_MOESM2_ESM.tif]
